# Mast Cell Association with the Microenvironment of a Phosphaturic Mesenchymal Tumour Secreting Fibroblast Growth Factor 23

**DOI:** 10.3390/medsci13030195

**Published:** 2025-09-16

**Authors:** Andrey Kostin, Alexei Lyundup, Alexander Alekhnovich, Aleksandra Prikhodko, Olga Patsap, Sofia Gronskaia, Zhanna Belaya, Olga Lesnyak, Galina Melnichenko, Natalia Mokrysheva, Igor Buchwalow, Markus Tiemann, Dmitrii Atiakshin

**Affiliations:** 1 RUDN University, 6 Miklukho-Maklaya St., 117198 Moscow, Russia; andocrey@mail.ru (A.K.); liundup-av@rudn.ru (A.L.); alekhnovich_av@pfur.ru (A.A.); prikhodko_at@pfur.ru (A.P.); olga@patsap.ru (O.P.); buchwalow@pathologie-hh.de (I.B.); 2 Endocrinology Research Center, Dm. Ulyanova St. 11, 117292 Moscow, Russia; gronskaya.sofia@endocrincentr.ru (S.G.); jannabelaya@gmail.com (Z.B.); teofrast2000@mail.ru (G.M.); mokrisheva.natalia@endocrincentr.ru (N.M.); 3 North-West State Medical University, 191015 Saint-Petersburg, Russia; olga.m.lesnyak@yandex.ru; 4 Institute for Hematopathology, Fangdieckstr. 75a, 22547 Hamburg, Germany; mtiemann@hp-hamburg.de

**Keywords:** phosphaturic mesenchymal FGF23-secreting tumour, tumour microenvironment, mast cells, tryptase, chymase, carboxypeptidase A3, spatial phenotyping, immune landscape, extracellular matrix, intercellular interaction, multiplex immunohistochemistry

## Abstract

**Background:** Phosphaturic mesenchymal tumours secreting fibroblast growth factor 23 (hereinafter referred to as FGF23+ PMT) are rare neoplasms that can cause hypophosphataemic osteomalacia, owing to excessive FGF23 production. Mast cells (MCs) play a key role in tumour biology by modulating proliferative activity of atypical cells, resistance to innate and acquired immunity, angiogenesis, and metastatic behaviour. However, MCs associated with FGF23+ PMT have not previously been investigated. This study, to our knowledge, is the first to characterise features of the tumour microenvironment through spatial phenotyping of the immune and stromal landscape, together with histotopographic mapping of intercellular MC interactions with other subcellular populations in FGF23+ PMT. **Methods:** Histochemical staining (haematoxylin and eosin, toluidine blue, Giemsa solution, picro-Mallory protocol, silver impregnation), as well as monoplex and multiplex immunohistochemical staining with spatial phenotyping, were performed to detect atypical FGF23-secreting cells, immune cells (CD3, CD4, CD8, CD14, CD20, CD38, CD68, or CD163), stromal components (CD31, α-SMA, or vimentin), and specific MC proteases (tryptase, chymase, or carboxypeptidase A3). Bioinformatics analysis using artificial intelligence technologies was applied for spatial profiling of MC interactions with tumour, immunocompetent, and stromal cells in the tumour microenvironment. **Results:** Bioinformatic analysis of the entire tumour histological section, comprising over 70,000 cells stained using monoplex and multiplex immunohistochemical protocols, enabled identification of more than half of the cell population. The most abundant were CD14+ (30.7%), CD163+ (23.2%), and CD31+ (17.9%) cells. Tumour-associated MCs accounted for 0.7% of the total pool of immunopositive cells and included both mucosal and connective tissue subpopulations, predominantly of the tryptase + chymase-CPA3-specific protease phenotype. This pattern reflected combined multidirectional morphogenetic processes in the patient’s FGF23+ PMT. More than 50% of MCs were colocalized with neighbouring cells of the tumour microenvironment within 20 μm, most frequently with monocytes (CD14+CD68+), M2 macrophages (CD68+CD163+), and endothelial cells (CD31+). In contrast, colocalization with atypical FGF23-secreting cells was rare, indicating minimal direct effects on tumour cell activity. Interaction with T lymphocytes, including CD8+, was also infrequent, excluding their activation and the development of antitumour effects. Mapping of MC histotopography validated the hypothesis of their inductive role in monocyte differentiation into M2 macrophages and probable polarisation of macrophages from M1 into M2, thereby contributing to slow tumour growth. MCs were further involved in extracellular matrix remodelling and participated in the formation of pro-osteogenic niches within the FGF23+ PMT microenvironment, leading to pathological osteoid development. **Conclusions:** This study demonstrated active MC participation in the evolution of the FGF23+ PMT microenvironment. The findings may be applied in translational medicine to develop novel algorithms for personalised therapy in patients with FGF23-secreting tumours, offering an alternative when surgical removal of the tumour is not feasible.

## 1. Introduction

Oncogenic osteomalacia is a rare disease caused by severe defects in bone mineralisation mechanisms, most commonly owing to excessive exposure to fibroblast growth factor-23 (FGF23) [1,2,3,4]. Oncogenic osteomalacia is most commonly caused by a phosphaturic mesenchymal tumour secreting FGF23 (hereinafter referred to as FGF23+ PMT), which usually shows benign features [5,6,7]. FGF23 inhibits phosphate reabsorption in the proximal convoluted tubule by inactivating the sodium-phosphate pump, leading to compensatory phosphate release from bone tissue and subsequent demineralisation [8,9,10]. FGF23+ PMT is frequently observed in middle-aged individuals and is characterised by severe bone and muscle tissue damage, often progressing to multiple fractures, muscle weakness, and severe pain syndrome [1,7]. Functional diagnostics using drugs that bind to somatostatin receptor subtype 2A, together with contrast-enhanced magnetic resonance imaging, represent the most accurate approaches for topical diagnostics [7]. To date, clinical manifestations, changes in laboratory parameters, and diagnostic methods for identifying FGF23+ PMT localization have been well studied and described [3,7,11,12]. However, descriptions of the pathohistological features of the disease remain scarce and fragmentary, being based primarily on histochemical analysis and lacking histoprofiling, spatial phenotyping, and bioinformatics evaluation of atypical, stromal, and immune cells, including mast cells (MCs) [1,11,13].

MCs are components of innate immunity, and their involvement in the initiation and pathogenesis of allergic diseases is well-documented [14]. In addition, MCs accumulate in most tumours, where their activation and migration are associated with either a favourable or poor prognosis depending on the tumour type [15,16]. Elevated levels of specific MC mediators are being investigated as potential markers for disease prognosis and chemotherapy monitoring [17,18,19,20]. MCs express a broad range of receptors for cytokines, chemokines, the complement system, and immunoglobulins, enabling them to respond rapidly and sensitively to various signals within the tumour microenvironment through the mediators they produce [21,22,23,24,25]. The influence of MCs on the tumour microenvironment is characterised by pronounced versatility for several reasons. MCs are major producers of biologically active substances that act directly on the tumour microenvironment. These mediators can be classified into three groups: preformed mediators, such as histamine, heparin, chymase, tryptase, and carboxypeptidase A3; de novo synthesized mediators, including leukotrienes and prostaglandins; and cytokines, including granulocyte-macrophage colony-stimulating factor, transforming growth factor-β (TGF-β), vascular endothelial growth factor (VEGF), basic FGF, matrix metalloproteinases (MMP), and histamine [23,26]. These mediators regulate intratumoural angiogenesis, tissue remodelling, metastasis, immune modulation, and the proliferative activity of tumour cells [27,28,29]. In particular, it was shown that the expression of MMPs was significantly correlated with the infiltration of various immune cells in breast cancer, including CD8+ and CD4+T cells, macrophages, neutrophils, B cells, and dendritic cells, suggesting the close correlations between MMPs and immune functions [30]. Among the MMPs, MMP-14 is the driving force behind extracellular matrix and tissue destruction during cancer invasion and metastasis [31]. Landscape analysis of tumor metabolic plasticity and landscape analysis of ferroptosis and cuproptosis-related genes are potential biomarkers for diagnosis, tumor classification and prognosis [32,33,34]. The role of MCs in shaping the tumour microenvironment, both pro-tumour and anti-tumorigenic, reflects the activity of their receptor repertoire, which determines the profile of secreted mediators in response to activating stimuli from cellular (immune, stromal, and tumour) or extracellular targets. In addition, MCs can exert direct cytotoxic or antiproliferative effects on atypical cells [35,36]. This broad spectrum of intercellular signalling explains why the consequences of MC accumulation vary across different tumour types [26,37,38,39,40].

To advance understanding of FGF23+ PMT pathogenesis, this study, to our knowledge, provides the first analysis of the tumour-associated MC population, with a specific focus on spatial phenotyping of MC integration into the immune and stromal landscapes of the tumour microenvironment in situ, as well as on intercellular interactions and the secretory pathways of specific proteases shaping the functional architecture of the tumour microenvironment.

## 2. Materials and Methods

### 2.1. Case Report

Biomaterials obtained from the inguinal region of a patient diagnosed with an FGF23-secreting soft tissue tumour were investigated.

The patient was a 21-year-old male with no prior symptoms, who began to experience severe pain in the back and hips three years before admission to the National Medical Research Centre of Endocrinology, Ministry of Health of the Russian Federation. As the pathology progressed, progressive muscle weakness was also observed. The study protocol was approved by the local ethics committee of the Russian National Research Centre of Endocrinology, Ministry of Health of the Russian Federation (protocol No. 17, dated 28 October 2020). Informed consent was obtained from all subjects involved in the study.

On examination, the patient was 184 cm tall and weighed 79 kg, with a body mass index of 23.3 kg/m^2^. The patient was an active athlete and reported no skeletal bone deformities or dental disease. Progressive muscle weakness resulted in a change in gait during the year prior to hospital admission. Laboratory tests revealed hypophosphatemia (0.45 mmol/L; reference range [RR] 0.74–1.52), elevated alkaline phosphatase level (472 U/L; RR 40–150), reduced tubular reabsorption of phosphate (62%; RR > 85%), and increased intact FGF23 (149 pg/mL), with normal parathyroid hormone (47 pg/mL) and albumin-corrected calcium (2.2 mmol/L). Serum iFGF23 levels were measured using an ELISA Kit (Biomedica BI-20700, Vienna, Austria); the median value in healthy individuals was 14.8 pg/mL (RR 3.8–25.0). Phosphate (RD 0.74–1.52 mmol/L), alkaline phosphatase (RD 40–150 IU/L), and parathyroid hormone (RD 15–65 ng/mL) levels were measured using the Abbott Architect c8000 analyser, Illinois, United States. Tubular phosphate reabsorption was calculated using the online tool (http://www.scymed.com/en/smnxps/pshpd274.htm, accessed on 9 September 2025). Radiographs demonstrated a significant decrease in bone mineral density parameters: Z-score −4.2 SD in the lumbar region and −3.2 SD in the femoral region.

**Figure 1:**
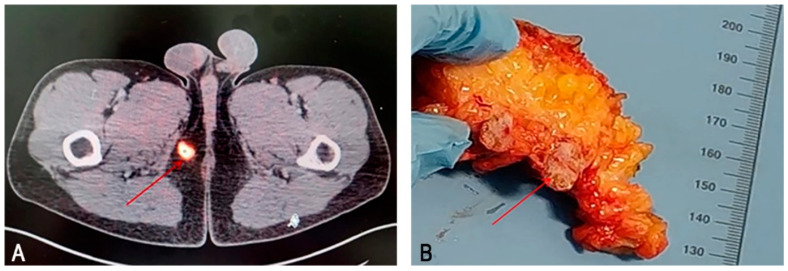
Phosphaturic mesenchymal tumour secreting fibroblast growth factor 23: positron emission tomography/computed tomography (PET/CT) and gross visualization images. (**A**) ^68^Ga-DOTATATE PET/CT showing a 2 cm soft tissue tumour, positive for somatostatin receptor, located in the subcutaneous adipose tissue of the inguinal region (arrow). (**B**) Gross specimen of the excised tumour (arrow).

Based on these findings, the patient was diagnosed with an FGF23-secreting soft tissue tumour of the inguinal region, accompanied by hypophosphataemic osteomalacia, bone pain, and muscle weakness associated with phosphorus deficiency. Oral phosphate supplementation and alfacalcidol (4 µg) therapy were initiated, leading to symptomatic relief of bone pain and an increase in phosphate levels to 0.66 mmol/L. Ga-DOTATATE positron emission tomography/computed tomography (CT) and CT identified a tumour located in the subcutaneous fat of the inguinal region under the inferior pubic branch, sized 18 × 12.5 × 14.8 mm (Figure 1A). The tumour was surgically excised (Figure 1B). Ten days after tumour removal, serum phosphate level was 1.21 mmol/L; after 3 months, it had risen to 1.54 mmol/L. Notably, bone pain was significantly reduced, and mobility increased within 6 months, indicating a sustained clinical recovery.

### 2.2. Sample Preparation

Following microscopy, the excised tumour was fixed in buffered formalin and processed according to standard sample preparation procedures. Histological sections 5 μm thick were prepared for histochemical staining, and histological sections 2.5 μm thick were prepared for monoplex and multiplex immunohistochemical staining protocols [41].

### 2.3. Immunohistochemistry (IHC) and Histochemistry

For IHC, deparaffinised sections underwent antigen retrieval by heating in a steamer with R-UNIVERSAL Epitope Recovery Buffer (Aptum Biologics Ltd., Southampton, SO16 8AD, UK) at 95 °C for 30 min. Blocking of endogenous Fc receptors prior to incubation with primary antibodies was omitted, in accordance with our earlier recommendations [42]. After antigen retrieval and, where required, quenching of endogenous peroxidase, sections were immunoreacted with primary antibodies. The list of primary antibodies used in this study is presented in Table 1. Immunohistochemical visualisation of bound primary antibodies was performed manually, following the standard protocol [41]. For manual immunostaining, primary antibodies were incubated overnight at +4 °C at the optimal dilution.

Bound primary antibodies were visualised using horseradish peroxidase (HRP)-conjugated secondary antibodies (Dianova, Hamburg, Germany; Molecular Probes, Darmstadt, Germany). The list of secondary antibodies and other reagents used in this study is presented in Table 2. Nuclei were counterstained with Mayer’s haematoxylin, and sections were mounted in a permanent medium.

Bound primary antibodies were visualised using secondary antibodies (Dianova, Hamburg, Germany; Molecular Probes, Darmstadt, Germany) conjugated with Alexa Fluor-488, Cy3, Alexa Fluor-555, Alexa Fluor-594, or Alexa Fluor-647 (Table 2 and Table 3). The final concentration of secondary antibodies was 5–10 µg/mL phosphate-buffered saline. Single and multiple immunofluorescence labelling were performed according to standard protocols [41]. Sequential multiplex immunohistochemical staining for the simultaneous detection of tryptase, chymase, carboxypeptidase A3 (CPA3), CD3, CD8, CD14, CD68, CD163, CD31, αSMA, and others (Table 2 and Table 3) was carried out following Akoya Biosciences (USA) recommendations for the use of OPAL series fluorochromes with the Mantra 2 Quantitative Pathology Imaging System (Table 2). In addition, for repeated retrieval when using OPAL series fluorochromes, the EZ-Retriever^®^ System MW015-IR (BioGenex, Fremont, CA, USA) was applied. Histochemical staining with toluidine blue, Giemsa solution, haematoxylin and eosin, picro-Mallory, and silver impregnation was performed according to the manufacturer’s instructions (Table 2).

Given that most MCs were tryptase-positive, this protease was used as the primary marker for studying MC co-localisation with other cells. Designs of duplex and multiplex immunohistochemical staining protocols are presented in Table 3.

### 2.4. Controls

Control incubations included samples without primary antibodies or with substitution of the primary antibodies by the same IgG species (Dianova, Hamburg, Germany) at the same final concentration as the primary antibodies. Exclusion of either the primary or secondary antibody from the immunohistochemical reaction, as well as substitution with corresponding IgG at the same final concentration, resulted in the absence of immunostaining. The specific and selective staining of different cell types using primary antibodies from the same species within the same preparation was considered sufficient control for immunostaining specificity.

### 2.5. Image Acquisition

For the study of microsections stained according to histochemical and immunohistochemical protocols, light microscopy was performed using a ZEISS AxioImager.Z2 microscope equipped with Zeiss alpha Plan-Apochromat objective 100×/1.46 OilDICM27, Zeiss Objective Plan-Apochromat 150×/1.35 GlycDICCorrM27, and ZEISS Axiocam 712 colour digital microscope camera and ZEISS Axiocam 712 mono digital microscope camera (Carl Zeiss Vision, Jena, Germany). In some cases, photomicrographs were obtained with a Nikon D-Eclipse C1 Si confocal microscope based on the Nikon Eclipse 90i platform (Nikon Instruments Inc., Tokyo, Japan). Multiplex visualisation was performed using the Mantra 2 Quantitative Pathology Imaging System (Akoya Biosciences, Marlborough, MA, USA), based on an Olympus BX43 microscope (Olympus Corporation, Tokyo, Japan) equipped with a scientific-grade multispectral 12-bit monochrome high-sensitivity CCD camera and a liquid crystal tunable spectral filter, to determine the profile of specific proteases of MCs and the immune and stromal landscape of the tumour microenvironment with different OPAL fluorochromes.

### 2.6. Quantitative Analysis

Quantitative analysis was performed on whole tumour sections. Planimetric analysis was used to determine the number of search cells (MCs, immune cells, stromal cells, and atypical tumour cells) per unit area of the sections, absolute and relative number of MCs and other cells, and profile of mast cell-specific proteases using the QuPath v0.5.1 software product [43]. Mapping and imaging of stained sections were performed after scanning the entire histological section using a digital pathology slide scanner (fluorescence) KF-FL-005 (Ningbo, Zhejiang, China), ×40 objective of a ScanScope CS (Leica Biosystems, Deer Park, IL, USA), and the Mantra 2 Quantitative Pathology Imaging System (Akoya Biosciences, Marlborough, MA, USA) based on an Olympus BX43 microscope. Nuclei were identified using the Stardist extension [44]. The intensity threshold for the DAPI channel was defined with a pixel classifier built into Qupath, trained on expert annotations. Further classification of segmented detections by phenotype was achieved by training a neural network with point annotations and subsequent iterative verification by a specialist. Autofluorescence was minimized using a dedicated reagent kit and by optimising exposure time during scanning. Cell co-localisation was calculated by measuring the distance between nuclei centres, taking into account the length of their semi-axes. The minimum distance, defined as the sum of the major semi-axes of the nuclei, averaged ≤10 μm. Distance categories were defined as follows: <10 μm, contacting cells; 10–20 μm, cells within the paracrine interaction zone; >20 μm, non-interacting cells. At each iteration, results were reviewed by a specialist and parameters adjusted as necessary. Quantitative data were exported from QuPath v0.6.0 to R for further visualisation and analysis [45].

## 3. Results

### 3.1. Histochemical Analysis

**Figure 2:**
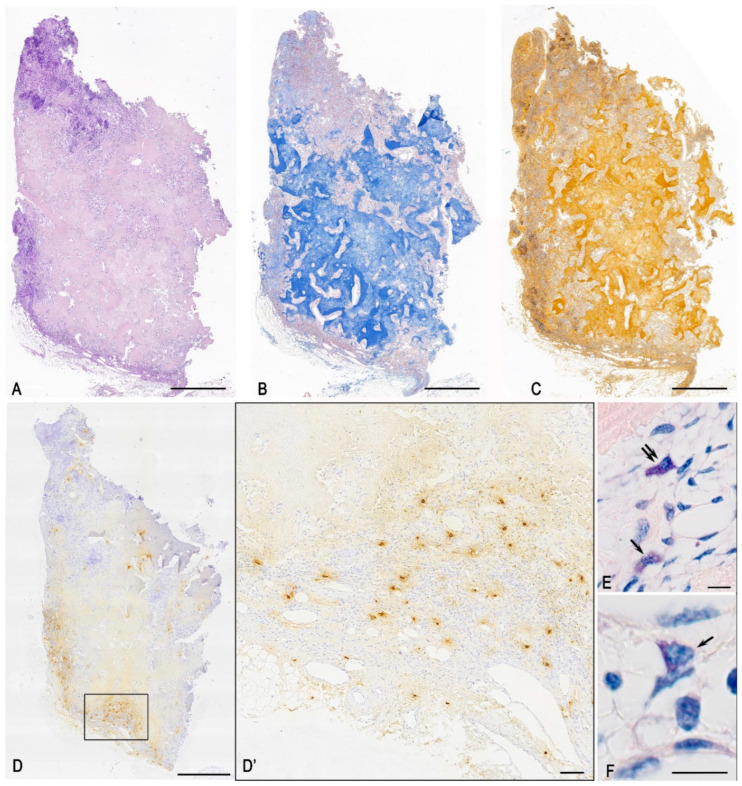
General view of the FGF23-secreting tumour. Techniques: (**A**–**C**) Histochemical staining with hematoxylin and eosin (**A**), picro-Mallory (**B**), Giemsa (**E**,**F**), and silver impregnation (**C**). (**D**) Immunohistochemical staining of mast cells (MCs) for tryptase. (**D’**) Enlarged fragment of (**D**), showing localisation of mast cells at the periphery of osteo-like formations. (**E**) Connective tissue MCs with high (arrow) and low (double arrowed) activity of secretory granules transfer into the extracellular matrix. (**F**) Small MCs with morphological features of the mucosal subpopulation (arrow). Scale bars: (**E**,**F**) = 10 μm; (**D’**) = 100 μm; others = 1000 μm.

**Figure 3:**
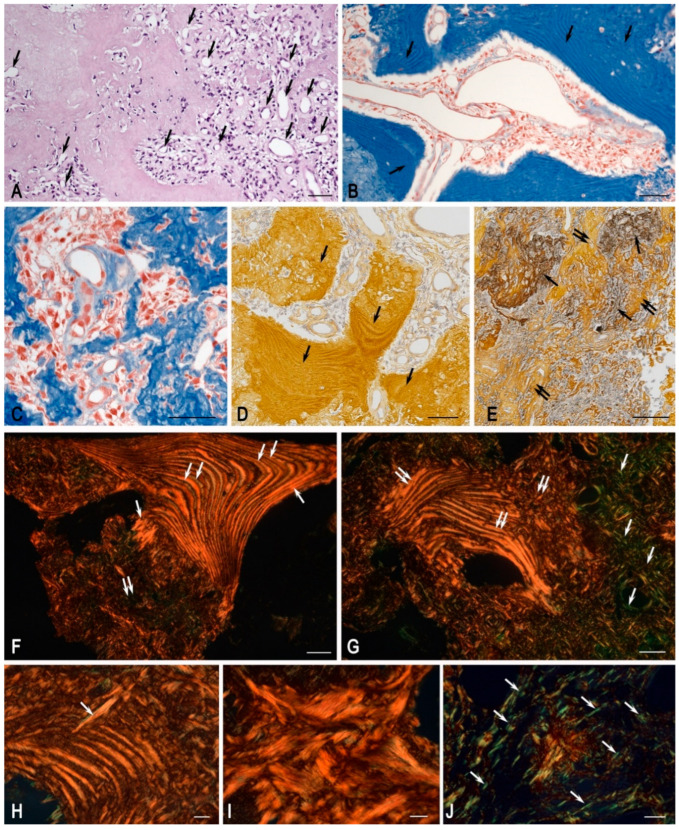
Features of osteo-like material and collagen fibrillogenesis in an FGF23-secreting tumour. Techniques: (**A**) Haematoxylin and eosin staining. (**B**,**C**) Picro-Mallory staining. (**D**,**E**) Silver impregnation. (**F**,**G**) Picrosirius red staining with polarisation microscopy. Notes: (**A**) Bone tissue with signs of sinus resorption of bone trabeculae and proliferation of loose connective tissue with vascular proliferation (arrow). (**B**,**D**) Pathological regeneration of bone tissue in the form of deformed layers of compact substance (arrow). (**C**) Locus of the tumour microenvironment with osteolytic outgrowths. (**E**) Tumour areas with high content of reticular fibers (arrow) and randomly oriented platelets of ossein fibrils with type I collagen in the background (double arrow). (**F**) Abnormal arrangement of osteoid as alternating bone plates with different directions of ossein fibrils and a predominant content of type I collagen (arrow). Minor areas of the tissue microenvironment rich in type III collagen, appearing as thin disordered fibers are found adjacent (double arrow). (**G**) Type I collagen in atypical plates of varying density and orientation of osteoid (double arrow) co-localising in the tumour microenvironment with extensive areas of type III collagen accumulation in thin, randomly oriented fibers (arrow). (**H**) Abnormal arrangement of bone plates, with one plate disrupting the spatial orientation of others (arrow). (**I**) Disordered arrangement of atypical bone plates with a predominant content of type I collagen within ossein fibrils. (**J**) Locus of the tumour microenvironment with chaotic arrangement of bone plates containing type III collagen within ossein fibrils (arrow). Scale bars: (**H**–**J**) = 10 μm; others = 50 μm.

**Figure 4:**
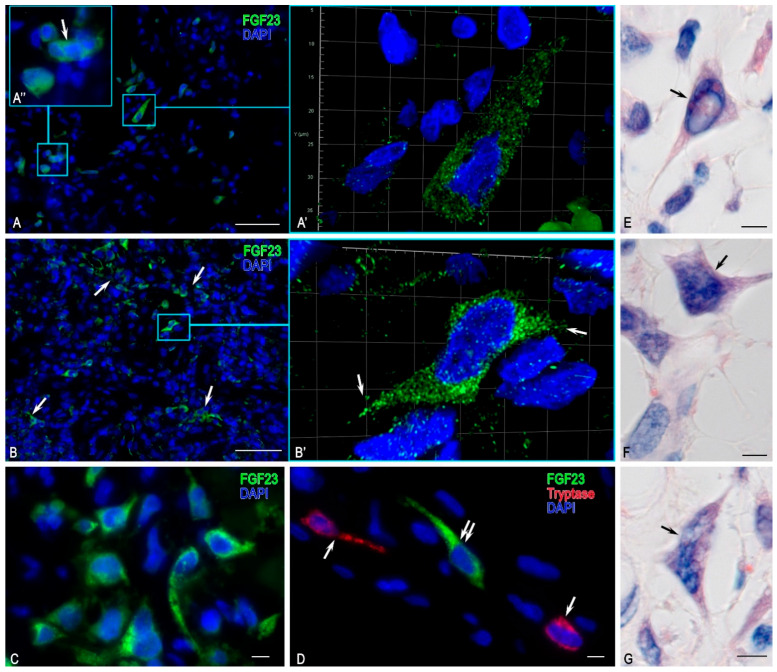
Morphology of FGF23+ cells in the tumour. (**A**) Diffuse arrangement of FGF23+ cells in the tumour. (**A’**) Three-dimensional (3D) model of an elongated FGF23+ cell (video presented in Supplementary S1). (**A”**) Enlarged view of (**A**) showing a likely multinucleated FGF23+ cell (arrow). (**B**) Formation of small islands of FGF23+ cells (arrow). (**B’**) 3D model of an enlarged (**B**) fragment showing an FGF23+ cell with outgrowths (arrow); video is presented in Supplementary S2. (**C**) Cluster of FGF23+ cells with variable morphology. (**D**) Co-localisation of two mast cells (arrow), one with a prolonged cytoplasmic outgrowth, together with an atypical FGF23+ tumour cell (double arrow). (**E**–**G**) Giemsa staining showing variants of atypical outgrowth-like cells (presumably, arrow). Scale bars: (**A**,**B**) = 50 µm; others = 5 µm.

In the examined tumour tissue sample, fibrous-vascular stroma, adipose tissue, bone tissue with signs of sinus resorption of bone trabeculae, proliferation of loose connective tissue with vascular proliferation, and a single osteoclast were identified. Pathological regeneration of bone tissue was observed in the form of deformed layers of compact substance; small, loosely arranged cells with round and oval nuclei were present between osteoid clusters (Figure 2A–C and Figure 3A,B,D,F–H). Staining according to the picro-Mallory protocol revealed a pathological osteoid structure comprising multidirectional soft plates with multiple cracks and cavities, accompanied by loci of osteolytic processes (Figure 2B and Figure 3B,C). Silver impregnation disclosed multiple osteo-like formations concentrated in the central region of the tumour specimen (Figure 2C and Figure 3D,E). Polarisation microscopy after picrosirius red staining demonstrated abnormal bone plates composed predominantly of type I collagen. These did not form classical osteons but instead produced functionless, chaotically arranged layers that impaired the mechanical strength of the structures (Figure 3F–J). Random orientation of bone plates was a consistent feature of all osteo-like formations; in some instances, certain plates were introduced into the hemispherical orientation of other plates at right angles (Figure 3H). Notably, both picrosirius red staining and silver impregnation of tumour tissues revealed a combination of osteolytic processes and the formation of new, immature collagen fibers. This was evidenced by loci of the local tissue microenvironment rich in reticular fibers, with randomly oriented plates of ossein fibrils in the background under silver impregnation, as well as thin collagen III-containing fibers under polarisation (Figure 3E,G,J). MCs were identified as both mucosal and connective tissue subpopulations, indicating complex multidirectional regulatory effects in the tumour microenvironment (Figure 2E,F). Giemsa staining further revealed cells whose shape and other morphological features resembled FGF23+ cells, suggesting that they represented an atypical population within this tumour (Figure 4E–G).

### 3.2. Monoplex and Multiplex Immunohistochemical Analysis

#### FGF23-Secreting Cells

Histological examination revealed FGF23+ cells with uneven distribution density, distinguished from surrounding cells by strong immunopositivity for this growth factor (Figure 4). FGF23-secreting cells were arranged in local clusters where they were either in direct contact with each other or within the paracrine interaction zone (Figure 4B). FGF23+ cells varied in size and morphology; most were round or spindle-shaped, with the spindle-shaped capable of forming outgrowths (Figure 4A,B). Three-dimensional (3D) modelling with confocal microscopy demonstrated cytoplasmic outgrowths in some cells. These cytoplasmic outgrowths extended both towards neighbouring FGF23+ cells and towards other cells of the tumour microenvironment. Some FGF23+ cells appeared multinucleated, although this may have been owing to dense cell co-localisation with each other (Figure 4). Notably, immunofluorescence analysis of the tumour stroma revealed immunopositive FGF23+ thin outgrowths extending in various directions and over different distances (Figure 4). Under diplex immunofluorescence detection of FGF23+ with tryptase, chymase, or CPA3, no direct contacts were detected between MCs and atypical FGF23+ cells. Only paracrine-distance co-localisation was observed (Figure 4D), suggesting the absence of immediate cytotoxic or modulatory effects of MCs on tumour cells in this patient.

### 3.3. Description of FGF23-Secreting Tumour-Associated MCs

**Figure 5:**
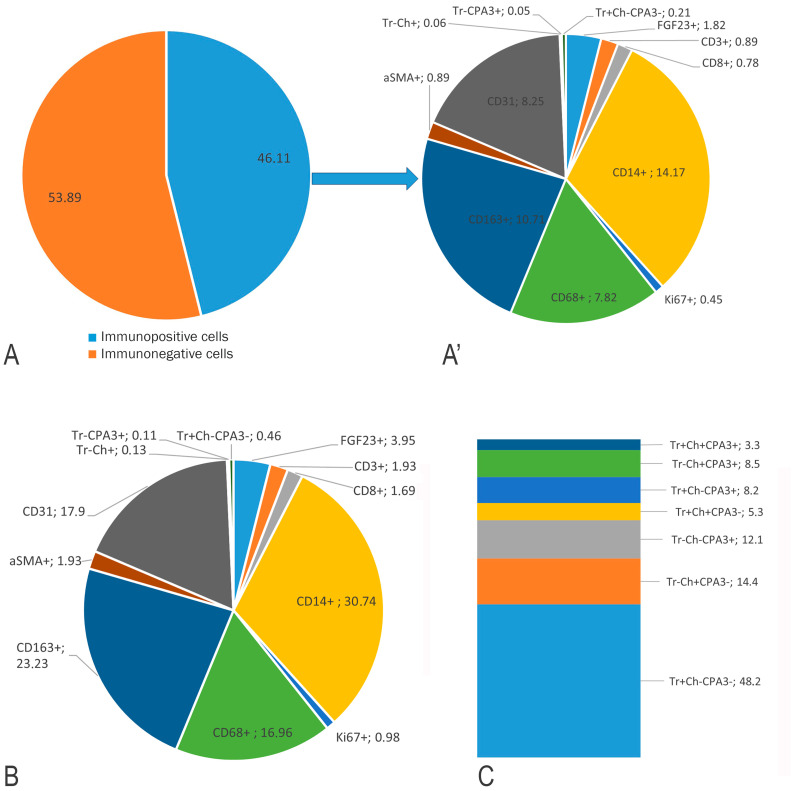
Profiles of immune and stromal cells in the FGF23-secreting tumour microenvironment. (**A**) Ratio of all phenotyped cells to other cells (%). (**A’**) Relative proportion of immunopositive cells to the total number of tumour cell populations (%). (**B**) Profiling of cell phenotypes within the total pool of immunopositive cells (%). (**C**) Profile of different phenotypes of tumour microenvironment-associated mast cells (%).

MCs constituted a minor population related to the FGF23-secreting tumour, representing 0.23% of all tumour cells and 0.7% of all immunopositive cells in whole-section phenotyping (Figure 5). In contrast, CD14+ monocytes and CD163+ and CD68+ macrophages predominated in the immune landscape of the FGF23-secreting tumour microenvironment, accounting for 30.74%, 23.23%, and 16.96% of all detectable immunopositive tumour cells, respectively (Figure 5). Endothelial cells comprised 17.9% of the total, reflecting a high degree of microvasculature in the tumour (Figure 5).

**Figure 6:**
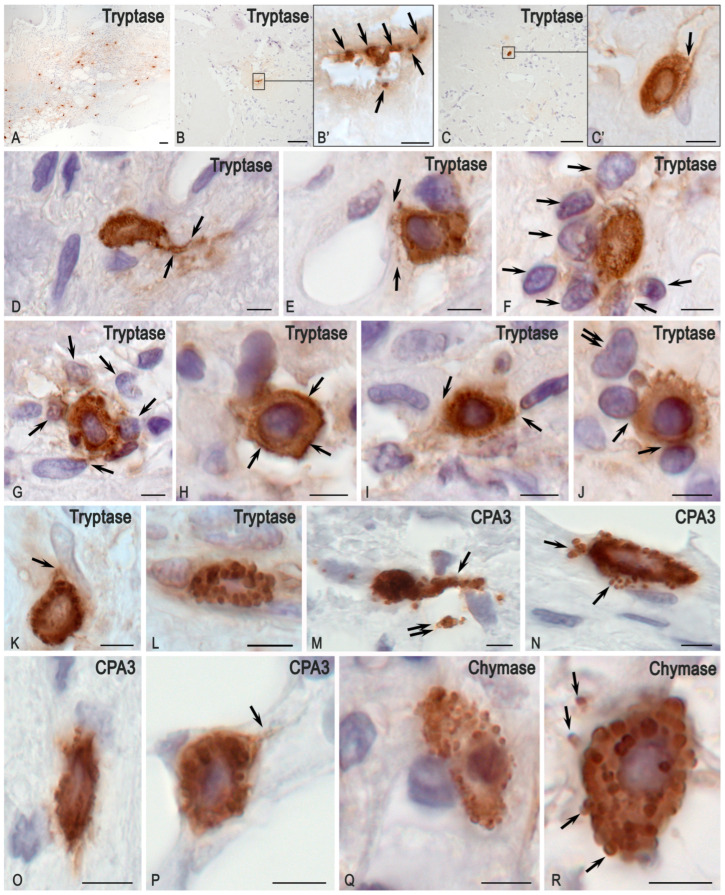
Mast cell (MC)-specific proteases in the regulation of the FGF23-secreting tumour microenvironment. (**A**) Predominant localisation of MCs at the tumour periphery. (**B**,**C**) Localisation of single MC and their derivatives in the central region of the FGF23+ PMT, in close contact with the osteo-like extracellular matrix. (**B’**,**C’**) Enlarged fragments of (**B**) and (**C**), respectively, showing active release of tryptase from autonomous MC granules ((**B’**), arrow) and from the MC via exosomal transport ((**C’**), arrow). (**D**) Active tryptase secretion spreading over paracrine distances to extracellular matrix targets (arrow). (**E**) Tryptase secretion to the basement membrane of capillary endothelial cells in the tumour microenvironment (arrow); its direction towards targets may be guided by nanotube formation (presumably). (**F**,**G**) Regulation of the tissue microenvironment by tryptase secretion simultaneously affecting several cells of the tumour microenvironment (arrow). (**H**) Use of transgranulation mechanism for tryptase secretion to targets in the local tissue microenvironment (arrow). (**I**) Interaction of MCs with stromal cells in the tumour microenvironment (presumably, arrow). (**J**) Effect of MC tryptase on lymphocytes of the tumour microenvironment (arrow) and on fibroblast (double arrow). (**K**) Contact of MCs with fibroblast during tryptase secretion (arrow). (**L**) Preferential localisation of tryptase in large secretory granules. (**M**) CPA3-positive granules localised in the cytoplasm of elongated MCs with a cytoplasmic outgrowth of considerable length (arrow). A large cytoplasmic fragment of the MC filled with granules is also visible (double arrow). (**N**) Secretion of individual large CPA3+ granules into the intercellular matrix (arrow). (**O**,**P**) Secretion of carboxypeptidase A3, which can be selectively transported to loci of the tissue microenvironment at paracrine distances (arrow). (**Q**,**R**) Variants of intracellular MC chymase accumulation in large secretory granules, subsequently secreted into the extracellular matrix (arrow). Scale bar: (**A**–**C**) = 50 μm; others = 5 μm.

Detection of MC profiles based on the expression of specific proteases revealed that approximately half exhibited the Tr+Ch-CPA3- phenotype, typical of the mucosal subpopulation (Figure 5C). MCs were characterized by the typical cytomorphology of enzymes with the accumulation of specific proteases, tryptase, chymase, and carboxypeptidase A3, primarily within secretory granules (Figure 6K–N,P–R). Histotopographically, MCs were located predominantly at the tumour periphery (Figure 2D and Figure 6A), although they were also observed in central tumour areas, where they actively interacted with the osteo-like extracellular matrix (Figure 6B,C). Morphologically, this interaction was expressed by signs of active secretion of specific MC proteases, tryptase, carboxypeptidase A3, and chymase, towards extracellular matrix targets (Figure 6B–D,K,L,Q,R). The predominant mechanisms of degranulation, documented by light, epifluorescence, and confocal microscopy, included the release of individual autonomous protease-positive secretory granules (Figure 6B,M,N,R), transgranulation (Figure 6H), exosome formation (Figure 6C,H–J), and the development of thin cytoplasmic outgrowths directed toward targets. These outgrowths, termed nanotubules, were visualized in small loci of the cytolemma not only by electron microscopy but also by confocal microscopy, and in large numbers by epifluorescence and light microscopy (Figure 6E).

Occasionally, MCs formed elongated thin outgrowths up to 0.5 μm in diameter, which may have served as conduits for carboxypeptidase A3 over paracrine distances (Figure 6P). A phenomenon was observed in which large autonomous cytoplasmic regions filled with granules detached from the maternal MC (Figure 6M,N); these detached regions were also capable of exerting selective effects on the tissue microenvironment over prolonged periods. Secretory granules located or transported into the extracellular matrix could release secretome components to molecular targets for some time (Figure 6B). In such cases, the duration of tryptase, chymase, or carboxypeptidase A3 secretion in the presence of appropriate stimuli was determined by the preformed intragranular level of enzymes. The secretion of MC-specific proteases to cellular and extracellular targets within the tumour microenvironment was predominantly targeted and could affect endothelial cells and the basement membrane of the microvasculature (Figure 6E), stromal cells, including fibroblasts and fibrocytes (Figure 6I–K), as well as various immunocompetent cells (Figure 6J). Notably, MCs often established large inductive fields simultaneously involving numerous cells of the tumour microenvironment that were affected by specific proteases (Figure 6F,G).

**Figure 7:**
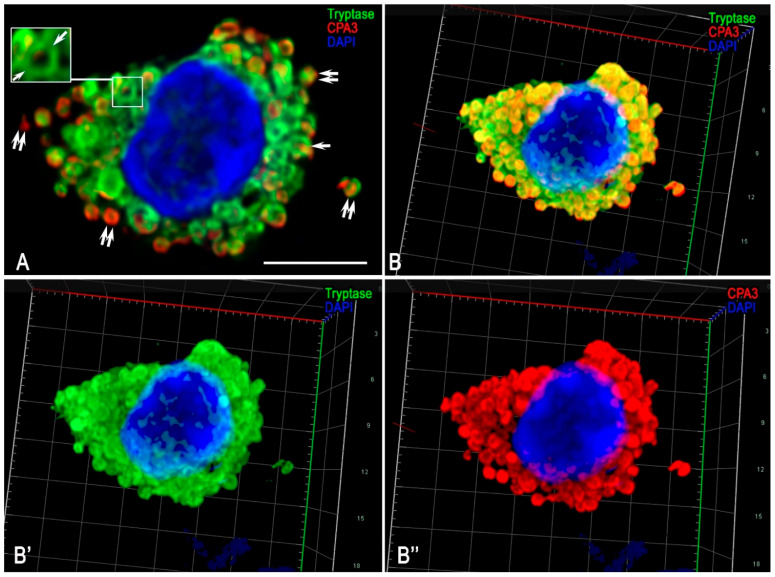
Cytotopography of tryptase and carboxypeptidase A3 in mast cells associated with FGF23-producing tumours. Technique: diplex immunohistochemical detection of tryptase (labelled with Alexa Fluor 488) and CPA3 (labelled with Alexa Fluor 594). (**A**) Predominant accumulation of specific proteases in secretory granules filling the cytoplasm, with peripheral intragranular co-localisation (arrow). Secretion of granules containing different levels of specific proteases is also observed (double arrow). (**B**) 3D models of the cytotopography of specific mast cell proteases in (**A**), with simultaneous (**B**) and separate (**B’**,**B”**) visualisation of tryptase and carboxypeptidase A3, are presented in Supplementary S3A, S3B, and S3C, respectively.

**Figure 8:**
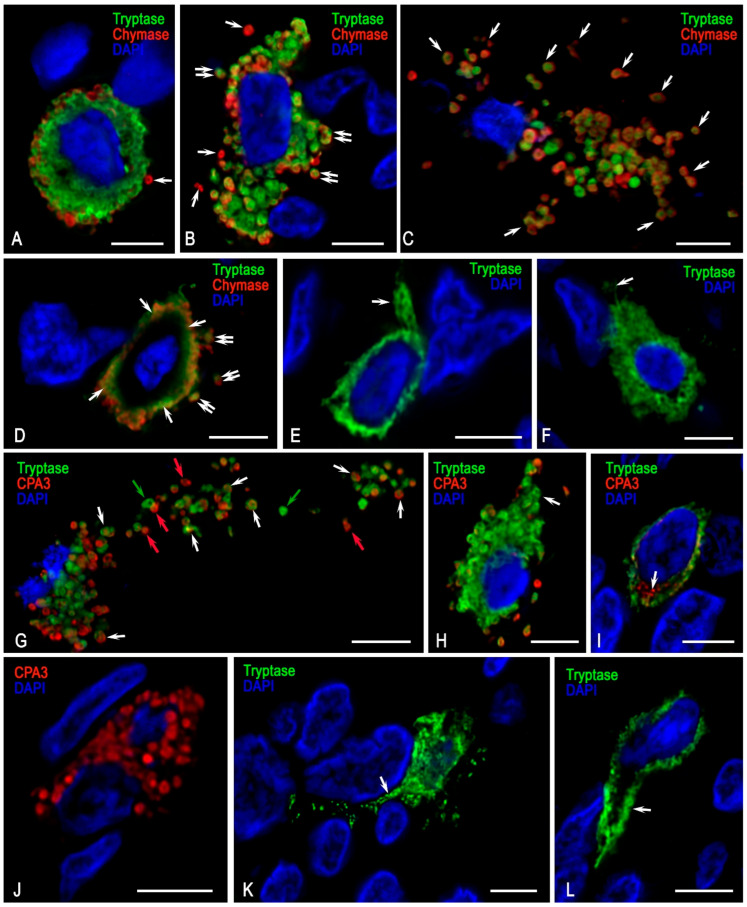
Visualisation of specific protease profiling in mast cells (MCs) associated with FGF23-producing tumour. Technique: (**A**–**F**) Diplex immunohistochemical detection of tryptase and chymase (labelled with Alexa Fluor 488 and Alexa Fluor 647, respectively), (**G**–**L**) diplex immunohistochemical detection of tryptase and CPA3 (labelled with Alexa Fluor 488 and Alexa Fluor 594, respectively). (**A**) A round MC with the Tryptase+Chymase+ phenotype secreting single chymase-positive granules (arrow). The 3D model is presented in Supplementary S4. (**B**) An elongated MC containing both tryptase and chymase. Active secretion of granules with the Tryptase-Chymase+ (arrow) and Tryptase+Chymase+ (double arrow) phenotypes is observed. The 3D model is presented in Supplementary S5. (**C**) Evidences of active MC degranulation with transfer of autonomous secretory granules (arrow) to the pathological osteoid region of the tumour microenvironment. The 3D model is presented in Supplementary S6. (**D**) Predominant mechanism of transgranulation in the delivery of tryptase and chymase to tumour microenvironment targets (arrow), with preserved secretion of individual granules (double arrow). The 3D model is presented in Supplementary S7. (**E**,**F**) MCs with the Tryptase+Chymase- phenotype (mucosal subpopulation) showing a small cytoplasm, with signs of forming large cytoplasmic outgrowths ((**E**), arrow) and nanotubules ((**F**), arrow) for the delivery of secretory products to tissue microenvironment targets. (**G**) Extensive MC degranulation with dissemination of secretory granules of various phenotypes in the area of pathological osteoid, including Tryptase+CPA3+ (white arrow), Tryptase+CPA3- (green arrow), and Tryptase-CPA3+ (red arrow). The 3D model is presented in Supplementary S8. (**H**) Tumour-associated MCs containing specific proteases in mature granules with signs of secretion into the extracellular matrix. (**I**) Tryptase and CPA3 localisation in immature secretory granules; CPA3 containing progranules are identified (arrow). (**J**) A MC with the Tryptase-CPA3+ phenotype; the cytoplasm is filled with granules containing carboxypeptidase A3. (**K**,**L**) MCs with the Tryptase-CPA3+ phenotype forming outgrowths of varying thickness and length to provide intercellular communication (arrow).

Profiling of specific MC proteases demonstrated high variability in the combinations of tryptase, chymase, and carboxypeptidase A3 within the secretome (Figure 7A,B). Notably, among tumour-associated MCs, the Tryptase+Chymase-CPA3- phenotype prevailed, accounting for more than half of the examined population. MCs expressing only tryptase represented a mucosal phenotype, characterized by small cell size (Figure 2F) and small tryptase-positive granules. Some MCs were able to form tryptase-immunopositive cellular outgrowths extending over significant distances, suggesting a specific mechanism of tumour microenvironment coordination mediated by tryptase. This morphology was clearly detected by confocal microscopy (Figure 8E,F,K,L). These findings indicate that tryptase, according to these cellular derivatives, can be directly transported to targets in the tumour microenvironment, including via nanotubes, thereby participating in direct cellular signalling, a phenomenon previously observed by electron microscopy of paragangliomas [36].

The connective tissue subpopulation of MCs was characterised by large cells predominantly containing large granules in the cytoplasm. Within these granules, a triad of specific proteases, tryptase, chymase, and CPA3, was identified simultaneously (Figure 7). 3D modelling clearly visualized the large size of the granules, with proteases localised at the periphery (Figure 7A, Supplementary S3A–C). During secretion and subsequent transfer within the tissue microenvironment, the peripheral intragranular localisation of proteases was preserved (Figure 7). Notably, diverse variants of specific MC protease secretion into the extracellular matrix were observed in association with granules. Secretory granules with the Tryptase+Chymase+ (Figure 8B–D, volumetric model in Supplementary S4), Tryptase+CPA3+ (Figure 8G,H), Tryptase-Chymase+ (Figure 8A,B, Supplementary S5), Tryptase-CPA3+ (Figure 8G,H,J, Supplementary S6–S8) phenotypes were distinguished. In addition, the process of biogenesis and maturation of secretory granules involved several stages of formation (the so-called types of secretory granules), during which the dynamics of the specific protease profile could be identified optically, culminating in the final qualitative and quantitative composition of intragranular mediators (Figure 8I).

Visualized results of histotopographic profiling of specific MC proteases associated with the FGF23-producing tumour demonstrated active granule secretion containing both tryptase and chymase, as well as secretion with chymase alone (Figure 8B, Supplementary S3A–C). In areas where the immune and stromal landscape contacted the zone of pathological osteoid, active degranulation was observed; in some cases, secretory granules were transferred over significant distances from the maternal cell (Figure 8C,G, Supplementary S5–S8). Some MCs used the secretory mechanism of transgranulation to regulate neighbouring cells; this process involved preferential localisation of secretory granules along the cell periphery and their colocalization with the cytoplasmic membrane (Figure 8D, Supplementary S7).

The results of spatial phenotyping of MC interactions with other representatives of the tumour microenvironment, including atypical FGF23-producing cells, revealed an almost complete absence of direct contact with tumour cells. Evidence was found only for paracrine exchange between MCs and tumour cells (Figure 4D).

**Figure 9:**
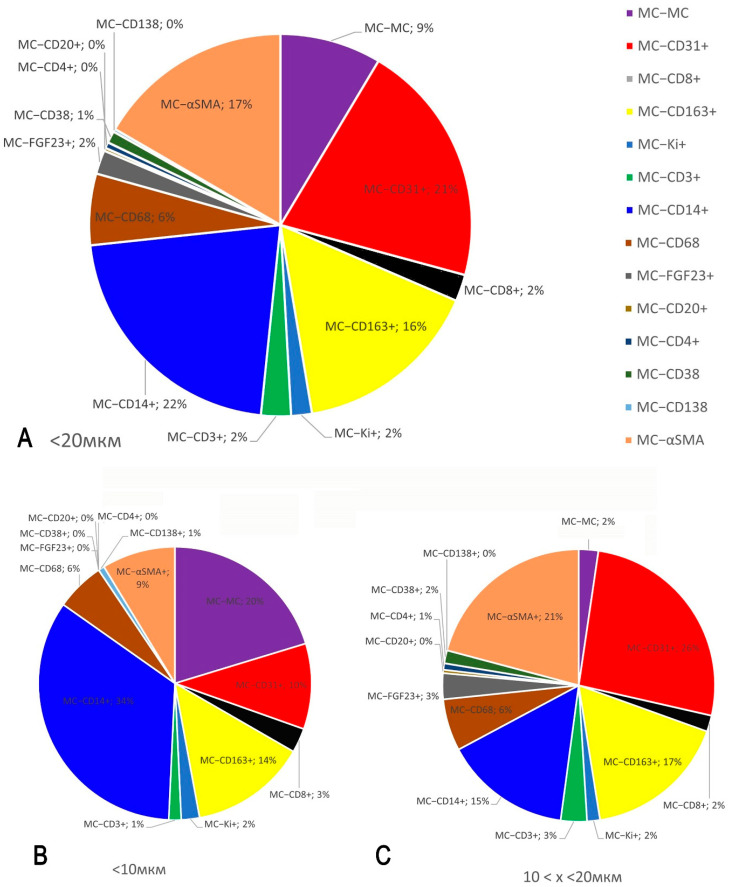
Spatial profiling of intercellular interactions (co-localisation) of tryptase-positive mast cells (MCs) with atypical, immunocompetent, and stromal cells of the FGF23-producing tumour microenvironment (in %, based on bioinformatics analysis of the entire section image). (**A**) Frequency of MC co-localisation with other cells of the FGF23-producing tumour microenvironment within the integral range of intertarget distances from 0 to 20 μm. (**B**,**C**) Frequency of MC co-localisation with other cellular targets of the tumour microenvironment within the ranges of 0 to 10 μm (**B**) and 10 to 20 μm (**C**). Explanations are provided in the text.

**Figure 10:**
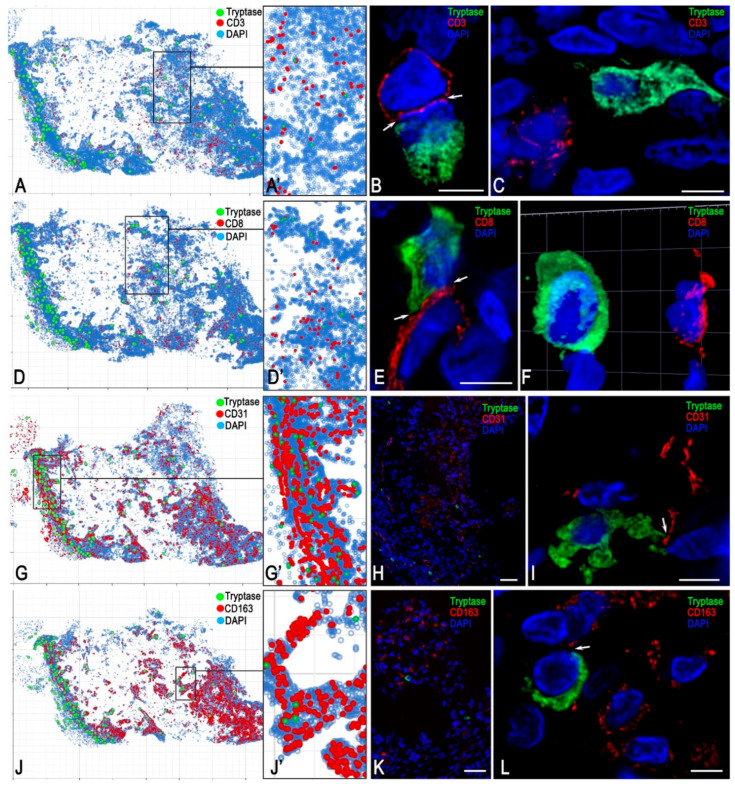
Mapping of tryptase-positive mast cells (MCs) in the immune and stromal landscape of the FGF23-producing tumour microenvironment. Technique: multiplex immunohistochemical staining of MC tryptase with CD3 (**A**–**C**), CD8 (**D**–**F**), CD31 (**G**–**I**), and CD163 (**J**–**L**). (**A**) Spatial phenotyping of MCs and T lymphocytes. (**A’**) Enlarged view of (**A**). (**B**,**C**) Juxtacrine ((**B**), arrow, 3D model presented in Supplementary S9) and paracrine (**C**) interactions of MCs and CD3+ lymphocytes. (**D**) Spatial phenotyping of MCs and cytotoxic T lymphocytes. (**D’**) Enlarged view of (**D**). (**E**) Direct contact between MCs and CD8+ lymphocytes (arrow). The 3D model is presented in Supplementary S10. (**F**) Paracrine co-localisation of MCs and T-killer. The 3D model is presented in Supplementary S11. (**G**) Spatial mapping of MCs and endothelial cells in the tumour microenvironment, showing intensive development of the microvasculature. (**G’**) Enlarged fragment of (**G**). (**G’**,**H**) Close MC co-localisation with endothelium. (**I**) Signs of tryptase secretion to the basement membrane and cytoplasm of endothelial cells (arrow). The 3D model is presented in Supplementary S12. (**J**) Histotopography of MC-M2 macrophage co-localisation. (**J’**) Enlarged fragment of (**J**). (**K**) High content of CD163+ macrophages in the tumour microenvironment. (**L**) Contact between MCs and M2 macrophages (arrow). The 3D model is presented in Supplementary S13. Scale bars: (**A**,**D**,**G**,**J**) = 1000 µm; (**H**,**K**) = 50 µm; others = 5 µm.

Spatial profiling of intercellular interactions, based on the results of mapping the immune and stromal landscape of the local tissue microenvironment, demonstrated juxtacrine and paracrine effects of tryptase-positive MCs on atypical, immunocompetent, and stromal cells associated with the FGF23-producing tumour. In particular, bioinformatics analysis of MC interaction with other cells of the FGF23-producing tumour microenvironment, within an integral range of intertarget distances from 0 to 20 μm, disclosed their most frequent co-localisation with CD14+, CD31+, αSMA+, and CD163+ cells. Notably, over 40% of MCs were in juxtacrine or paracrine contact with the monocyte-phagocytic link of innate immunity; of these, 22% were in contact with monocytes and 16% with M2-type macrophages (Figure 9A and Figure 10J–L). Intrapopulation contacts between MCs, as well as with T lymphocytes, were observed less frequently (Figure 10A–F).

As detected, when ranking MC co-localisation with other cellular phenotypes of the tumour microenvironment within the ranges of 0–10 and 10–20 μm, the most frequent juxtacrine interactions involved intercellular signalling between MCs themselves and with CD14+ monocytes (Figure 9B), whereas contacts with the endothelium were less frequent (9%). Conversely, paracrine interactions of MCs prevailed with microvasculature components, accounting for 26% and 31% of contacts with CD31+ and αSMA+ cells, respectively (Figure 9C and Figure 10G–I). Notably, the tumour microenvironment demonstrated a high density of endothelial cells (Figure 10G,G’,H).

## 4. Discussion

The histological characterisation of PMT remains limited, being based predominantly on classical haematoxylin and eosin staining. Histological features of these tumours are characterized by spindle-shaped cells, multinucleated giant cells, and calcifications embedded in a chondromyxoid or osteoid matrix, together with fibroblast-like cells, variable cell density, and “rough” calcification [1,11,13]. Immunohistochemical analysis in previous studies has demonstrated that atypical cells are immunopositive for vimentin, SATB2, smooth muscle actin, SSTR2, CD56, SATB2, and caldesmon, whereas being immunonegative for S-100, CD68, desmin, CD34, and cytokeratin [11,46,47]. Osteoblasts and osteocytes can act as FGF23 producers [5,7]. Recently, particular attention has focused on mesenchymal stromal cells, which retain the potential for multilineage differentiation [12,48]. Tumours enriched in mesenchymal stem cells are unique in both their properties and manifestations [49]. In such cases, osteomalacia is often the predominant manifestation, reflecting the direct effects of these tumours on bone [50].

The present study demonstrated that a number of spindle-shaped cells are FGF23+ and may serve as the structural and functional basis of tumour formation. This technology can be regarded as an important addition to the determination of FGF23 (phosphatonin) in the blood, alongside clinical and microscopic findings [51]. As reported previously, mitotic figures in these tumours are rare, although areas with red blood cell extravasation have been observed [52,53]. The apparently low proliferative activity may account for the slow growth and small size of these tumours, which complicates early detection; consequently, localisation of the tumour may take several years. In some cases, spindle cell proliferation was identified without evidence of cytological atypia [13]. The present findings on mitotic activity profiling support the conclusions of these earlier studies [13].

The aetiology and pathogenesis of FGF23-positive PMTs remain unknown [12]. Although the majority of PMTs are benign neoplasms, relapses have occasionally been reported, accompanied by malignant features such as nuclear atypia, multicellularity, increased mitotic activity, and the development of metastases [1,54,55,56,57]. This underlines the importance of investigating the pathogenesis of FGF23+ PMTs, particularly with respect to the active participation of MCs.

This study is the first to analyse MCs associated with an FGF23-producing tumour, including spatial phenotyping of specific proteases, tryptase, chymase, and carboxypeptidase A3. The integration of tryptase-positive MCs into the immune and stromal landscape of the tumour microenvironment was analyzed. MCs were generally small, measuring 8–12 μm, and most displayed a predominantly rounded cytoplasmic morphology. However, some of the MCs exhibited an elongated form with developing outgrowths. Proteases were predominantly localised within granules; however, in some cells no marked granule accumulation was observed, with proteases instead accumulating in the intergranular cytoplasmic matrix.

Notably, total mapping of MC interaction with other tumour cell populations was performed for the first time, accompanied by 3D model construction of juxtacrine and paracrine co-localisation. Furthermore, spatial phenotyping of the tumour microenvironment using multiplex immunohistochemistry technology with subsequent bioinformatics analysis enabled spatial profiling of the immune and stromal landscape of the tumour microenvironment, thereby identifying the prevailing immunophenotypes. In addition, immunohistochemical histotopographic analysis revealed uneven distribution patterns of FGF23-producing cells of varying morphologies within tumour tissue, forming high-density loci that appear to constitute the structural basis for intratumoural contact and interaction. Previous attempts to identify tumour cells employed molecular biological methods, including in situ hybridization, which pronounced diffuse cytoplasmic reactivity of FGF23 in the cytoplasm of neoplastic cells [13,58]. However, these technologies failed to provide a comprehensive depiction of the morphology and structural characteristics of atypical cells. In the present study, 3D models of atypical cells of an FGF23-producing tumour in situ were obtained for the first time using confocal microscopy of sections immunohistochemically stained with antibodies to FGF23. No evidence of juxtacrine interaction and only weak paracrine interaction between FGF23+ atypical cells and MCs was observed. These findings suggest the absence of direct cytotoxic effects of specific MC proteases on atypical cells, with their role more likely confined to indirect modulation of the functional architecture of an FGF23-producing tumour microenvironment.

MCs exert both antitumour and protumour functions during tumour progression [59,60,61,62,63]. Protumour MC activity is mediated through the secretion of a wide range of angiogenic factors, including specific proteases, MMPs, FGF-2, VEGF, and interleukin-8. Conversely, tumour growth can be suppressed by other cytokines and growth factors such as tumour necrosis factor-alpha (TNF-α), TGF-β, interferon-alpha, bioactive monoamines, and through the antiproliferative effects of tryptase, which have been documented in melanoma [35,37,39]. MCs have also been shown to exert a protective role at the early stages of intestinal oncogenesis [64]. In this context, MC polarisation may occur in a manner analogous to the functional status described for macrophages (M1 and M2) and neutrophils (N1 and N2) [39]. Consequently, MCs contribute to the formation of an immunosuppressive microenvironment that prevents cytotoxic reactions of natural killer (NK) and T cells against tumour cells [65].

It is crucial to consider MC interactions with myeloid-derived suppressor cells [66] and regulatory T cells [67], as well as their capacity to enhance antitumor activity through the recruitment and activation of T cells, NK cells, and dendritic cells [68,69]. Tryptase, chymase, and carboxypeptidase A3, characterised by selective secretion to targets within the tissue microenvironment, can exert multifaceted effects on tumour development [39,70]. The significant role of MCs is further underscored by their expression of MHC class II molecules, which confer antigen-presenting ability and enable participation in T-cell activation [71]. Moreover, MCs play a key role in T-cell activation, particularly through the expression of TNF and other co-stimulatory molecules that promote T-cell initiation and proliferation [72]. However, the present findings of only rare MC interactions with CD3 and CD8 lymphocytes suggest the absence of this antitumour mechanism in FGF23+ PMT. In addition, MCs can affect the maturation and development of B cells through the secretion of various cytokines, including IL-4, IL-5, IL-6, and IL-13 [73]. However, the almost complete absence of B cells in the tumour microenvironment excludes their activation by MCs, which normally occurs through direct intercellular interactions during the developing antitumor immunogenesis [74]. By contrast, the high percentage of MCs interacting with CD14+CD68+ monocytes is noteworthy. Macrophage profiling revealed the prevalence of the M2-type macrophage subpopulation over the M1 type, a finding that likely contributes to tumour course. Notably, some studies reported that CD68+ cells were not identified in PMT [46,47].

## 5. Conclusions

The interaction of MCs with monocytes appears to promote their polarisation into the anti-inflammatory M2 macrophage phenotype, accompanied by enhanced angiogenesis. This hypothesis is supported by the high proportion of CD31-immunopositive cells in the tumour microenvironment. Moreover, the active secretion of tryptase into the extracellular matrix exhibits powerful angiogenic effects, partly by stimulating capillary endothelial cell proliferation, promoting reformation of vascular structures, and remodelling of the extracellular matrix to establish local tissue niches for the development of tumour blood vessels. Histamine, closely associated with tryptase, chymase, and carboxypeptidase A3 in MC granules, is also likely released into the extracellular matrix during secretion and exerts proangiogenic properties that promote the formation of tumour blood vessels [23,28,75].

The obtained data reveal novel mechanisms of FGF23+ PMT development involving MCs and may be used in translational medicine by supporting the development of new algorithms for personalized therapies as alternatives to surgical treatment when tumour removal is not feasible [76].

## Figures and Tables

**Table 1:** Primary antibodies used in this study.

Antibodies	Host	Catalogue Number	Dilution	Source
Tryptase	Mouse monoclonal	ab2378	1:4000	AbCam, Cambridge, UK
Chymase	Mouse monoclonal	ab2377	1:5000	AbCam, Cambridge, UK
Anti-FGF 23	Goat Polyclonal	ab56326	1:1000	AbCam, Cambridge, UK
CPA3	Rabbit Polyclonal	ab251696	1:500	AbCam, Cambridge, UK
CD3	Rabbit monoclonal	ab16669	1:150	AbCam, Cambridge, UK
CD8	Rabbit monoclonal	ab101500	1:100	AbCam, Cambridge, UK
CD31	Rabbit monoclonal	ab182981	1:2000	AbCam, Cambridge, UK
CD14	Rabbit monoclonal	EPR3653	RTU	Cell Marque, Rocklin, CA, USA
CD68	Mouse monoclonal	ab955	1:3000	AbCam, Cambridge, UK
CD138	Mouse monoclonal	B-A38	RTU	Cell Marque, Rocklin, CA, USA
CD163	Rabbit monoclonal	ab182422	1:500	AbCam, Cambridge, UK
Ki67	Mouse monoclonal	ab279653	1:1000	AbCam, Cambridge, UK
HEXAb	Rabbit monoclonal	ab140649	1:200	AbCam, Cambridge, UK
CD4	Rabbit Multiclonal	ab288724	1:1000	AbCam, Cambridge, UK
CD20	Rabbit monoclonal	ab166865	1:200	AbCam, Cambridge, UK
CD38	Rabbit monoclonal	ab108403	1:500	AbCam, Cambridge, UK
Vimentin	Rabbit monoclonal	ab92547	1:500	AbCam, Cambridge, UK
αSMA	Mouse monoclonal	ab7817	1:5000	AbCam, Cambridge, UK

**Table 2:** Secondary antibodies and other reagents were used in this study.

Antibodies and Other Reagents	Source	Dilution	Label
Goat anti-mouse IgG Ab (#ab97035)	AbCam, Cambridge, UK	1/300	Cy3
Goat anti-rabbit IgG Ab (#ab150077)	AbCam, Cambridge, UK	1/300	Alexa Fluor 488
Goat Anti-Rabbit IgG H&L (Alexa Fluor^®^ 555) (#ab150078)	AbCam, Cambridge, UK	1/300	Alexa Fluor 555
Goat Anti-Mouse IgG H&L (Alexa Fluor^®^ 555) preadsorbed (#ab150118)	AbCam, Cambridge, UK	1/300	Alexa Fluor 555
Goat Anti-Mouse IgG H&L (Alexa Fluor^®^ 594) (#ab150116)	AbCam, Cambridge, UK	1/200	Alexa Fluor 594
Goat Anti-Mouse IgG H&L (Alexa Fluor^®^ 647) (#ab150115)	AbCam, Cambridge, UK	1/200	Alexa Fluor 647
Goat Anti-Rabbit IgG H&L (Alexa Fluor^®^ 647) (#ab150079)	AbCam, Cambridge, UK	1/200	Alexa Fluor 647
DAPI FP1501001KT	Akoya Biosciences, Marlborough, MA, USA	ready-to-use	DAPI
1X Plus Auto Amplification Diluent FP1609	Akoya Biosciences, Marlborough, MA, USA	ready-to-use	w/o
Opal Polymer HRP Ms + Rb Akoya Biosciences ARH1001EA	Akoya Biosciences, Marlborough, MA, USA	ready-to-use	HRP
Antibody Diluent/Block buffer Akoya Biosciences ARD1001EA	Akoya Biosciences, Marlborough, MA, USA	ready-to-use	w/o
Secondary antibodies conjugated with horseradish peroxidase FP1500001KT	Akoya Biosciences, Marlborough, MA, USA	ready-to-use	Opal 480
Secondary antibodies conjugated with horseradish peroxidase Opal 520 Reagent (#FP1487001KT)	Akoya Biosciences, Marlborough, MA, USA	ready-to-use	Opal 520
Secondary antibodies conjugated with horseradish peroxidase Opal 540 Reagent (#FP1494001KT)	Akoya Biosciences, Marlborough, MA, USA	ready-to-use	Opal 540
Secondary antibodies conjugated with horseradish peroxidase Opal 570 Reagent (#FP1488001KT)	Akoya Biosciences, Marlborough, MA, USA	ready-to-use	Opal 570
Secondary antibodies conjugated with horseradish peroxidase Opal 620 Reagent (#FP1495001KT)	Akoya Biosciences, Marlborough, MA, USA	ready-to-use	Opal 620
Secondary antibodies conjugated with horseradish peroxidase Opal 650 Reagent (#FP1496001KT)	Akoya Biosciences, Marlborough, MA, USA	ready-to-use	Opal 650
Secondary antibodies conjugated with horseradish peroxidase Opal 690 Reagent (#FP1497001KT)	Akoya Biosciences, Marlborough, MA, USA	ready-to-use	Opal 690
Secondary antibodies conjugated with horseradish peroxidase Opal 780 Reagent Pack (FP1501001KT)	Akoya Biosciences, Marlborough, MA, USA	ready-to-use	Opal 780
AmpliStain^TM^ anti-Mouse 1-Step HRP (#AS-M1-HRP)	SDT GmbH, Baesweiler, Germany	ready-to-use	HRP
AmpliStain^TM^ anti-Rabbit 1-Step HRP (#AS-R1-HRP)	SDT GmbH, Baesweiler, Germany	ready-to-use	HRP
4′,6-diamidino-2-phenylindole (DAPI, #D9542-5MG)	Sigma, Hamburg, Germany	5 µg/mL	w/o
VECTASHIELD^®^ Mounting Medium (#H-1000)	Vector Laboratories, Burlingame, CA, USA	ready-to-use	w/o
DAB Peroxidase Substrate Kit (#SK-4100)	Vector Laboratories, Burlingame, CA, USA	ready-to-use	DAB
Toluidine blue (Biovitrum, #07-002)	ErgoProduction LLC, Saint Petersburg, Russia	ready-to-use	w/o
Giemsa solution (Biovitrum, #21-023)	ErgoProduction LLC, Saint Petersburg, Russia	ready-to-use	w/o
Silver impregnation (Biovitrum, #21-026)	ErgoProduction LLC, Saint Petersburg, Russia	ready-to-use	w/o
Weigert–Van Gieson (Biovitrum, #21-020)	ErgoProduction LLC, Saint Petersburg, Russia	ready-to-use	w/o
Picro Sirius Red Stain Kit (Connective Tissue Stain) (#ab150681)	AbCam, Cambridge, United Kingdom	ready-to-use	w/o
Mayer’s Haematoxylin (Biovitrum, #05-002)	ErgoProduction LLC, Saint Petersburg, Russia	ready-to-use	w/o

**Table 3:** Designs of duplex and multiplex immunohistochemical staining.

Design Challenge	Detection Targets	Label	Counterstaining of Nuclei
Profile of MCs specific proteases	Tryptase + Chymase	Alexa Fluor 488 and Alexa Fluor 647	DAPI (Sigma, Hamburg, Germany)
	Tryptase + CPA3		
	Chymase + CPA3		
	Tryptase + Chymase + CPA3	OPAL 480, 540 and 690	DAPI (Akoya Biosciences, Marlborough, MA, USA)
Interaction of MCs with atypical cells	Tryptase + FGF23	Alexa Fluor 488 and Alexa Fluor 647	DAPI (Sigma, Hamburg, Germany)
Immune landscape of the tumour microenvironment	Tryptase + CD3	Alexa Fluor 488 and Cy3	
	Tryptase + CD4	Alexa Fluor 488 and Cy3	
	Tryptase + CD8	Alexa Fluor 488 and Cy3	
	Tryptase + CD14	Alexa Fluor 488 and Cy3	
	Tryptase + CD20	Alexa Fluor 488 and Cy3	
	Tryptase + CD38	Alexa Fluor 488 and Cy3	
	Tryptase + CD68	Alexa Fluor 488 and Cy3	
	Tryptase + CD138	Alexa Fluor 488 and Cy3	
	Tryptase + CD163	Alexa Fluor 488 and Cy3	
Stromal landscape of the tumour microenvironment	Tryptase + CD31	Alexa Fluor 488 and Cy3	
	Tryptase + aSMA	Alexa Fluor 488 and Cy3	
Proliferative activity	Tryptase + Ki67	Alexa Fluor 488 and Cy3	
Immune and stromal landscape	Tryptase + CD3+ CD8+ CD14 + CD68+ CD163 + CD31+ aSMA	OPAL 480, OPAL 520, OPAL 540, OPAL 570, OPAL 620, OPAL 650, OPAL 690, OPAL 780	DAPI (Akoya Biosciences, Marlborough, MA, USA)

## Data Availability

The original contributions presented in this study are included in the article/Supplementary Material. Further inquiries can be directed to the corresponding authors.

## Supplementary Materials

The following supporting information can be downloaded at: https://www.mdpi.com/article/10.3390/medsci13030195/s1, Supplementary S1: 3D model of the FGF-23+ elongated cell title; Supplementary S2: 3D model of the enlarged FGF23+ of the cell with outgrowths; Supplementary S3A: 3D model of the cytotopography of specific mast cell proteases with simultaneous content of tryptase (green color) and carboxypeptidase A3 (red color): joint visualization; Supplementary S3B: 3D model of the cytotopography of specific mast cell proteases with simultaneous content of tryptase and carboxypeptidase A3: volumetric visualization of the intracellular localization of tryptase (green color); Supplementary S3C: 3D model of the cytotopography of specific mast cell proteases with simultaneous content of tryptase and carboxypeptidase A3: volumetric visualization of the intracellular localization of carboxypeptidase A3 (red color); Supplementary S4: 3D model of a round mast cell with the Tryptase+Chymase+ phenotype secreting single chymase-positive granules. Tryptase is green, chymase is red; Supplementary S5: 3D model of an elongated mast cell containing both tryptase (green) and chymase (red). Active secretion of granules with the Tryptase–Chymase+ and Tryptase+ Chymase+ phenotypes is observed; Supplementary S6: 3D model of active mast cell degranulation features with transfer of autonomous secretory granules (arrowed) into the pathological osteoid area of the tumor microenvironment. Tryptase is green, chymase is red; Supplementary S7: The predominant mechanism of transgranulation in the tryptase (green) and chymase (red) delivery to tumor microenvironment targets, with preserving secretion of individual granules into the extracellular matrix; Supplementary S8: 3D model of pronounced mast cell degranulation with dissemination of secretory granules of various phenotypes in the area of pathological osteoid, including Tryptase+CPA3+, Tryptase+CPA3- and tryptase-CPA3+. Tryptase is green, CPA3 is red; Supplementary S9: 3D model of juxtacrine interaction between mast cells (green) and CD3+ lymphocytes (red); Supplementary S10: 3D model of MC (green) and CD8+ lymphocyte (red) direct contacting; Supplementary S11: 3D model of paracrine colocalization of a MC (green color) and a T-killer (red color); Supplementary S12: 3D model of close MC colocalization (green) with endothelium (red) and signs of tryptase secretion to the basement membrane and cytoplasm of endothelial cells; Supplementary S13: 3D model of contacting between MC (green) and M2 type macrophage (red).
