# Supplementary material for: Mast Cell Association with the Microenvironment of a Phosphaturic Mesenchymal Tumour Secreting Fibroblast Growth Factor 23
Source: Med Sci (Basel). 2025 Sep 16;13(3):195. doi: 10.3390/medsci13030195 (PMC12452509; doi:10.3390/medsci13030195)

SUPPLEMENTARY MATERIALS

Supplementary 1

3D model of the FGF-23+ elongated cell.

https://disk.yandex.ru/d/J3-sEo-H8sZOyA

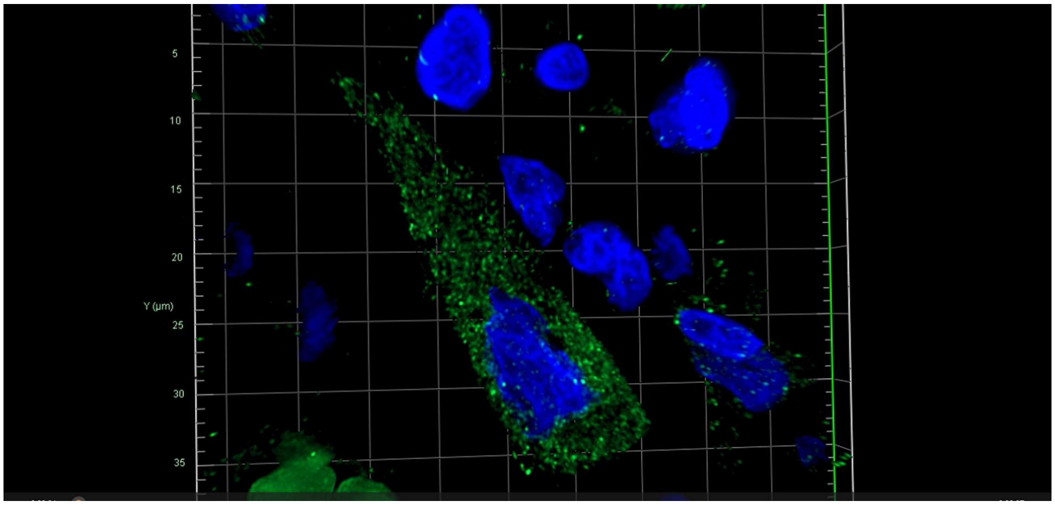

Supplementary 2

3D model of the enlarged FGF23+ of the cell with outgrowths.

https://disk.yandex.ru/i/BjxHv2CkpiAHtg

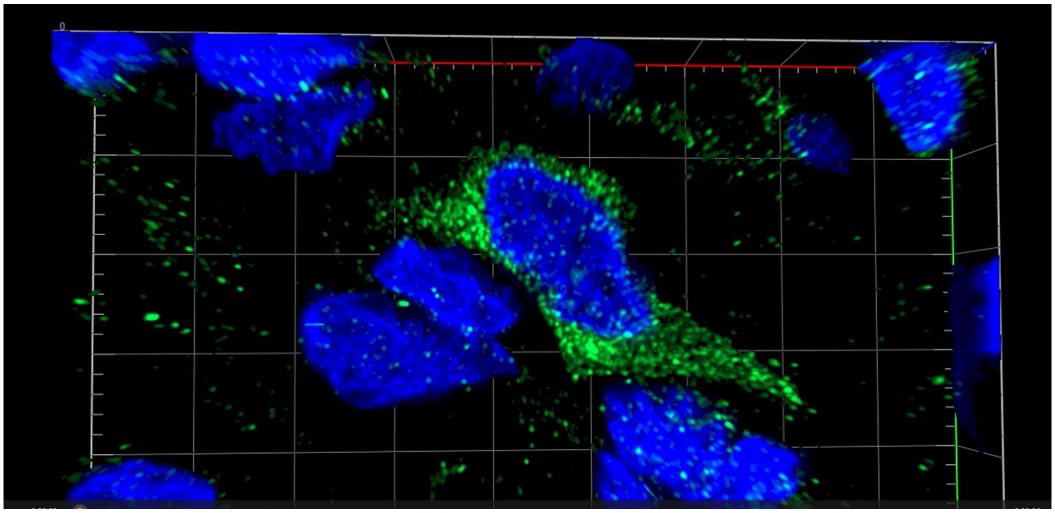

Supplementary 3A

3D model of the cytotopography of specific mast cell proteases with simultaneous content of tryptase (green color) and carboxypeptidase A3 (red color): joint visualization.

https://disk.yandex.ru/d/NqpBhkCCCIfQsw

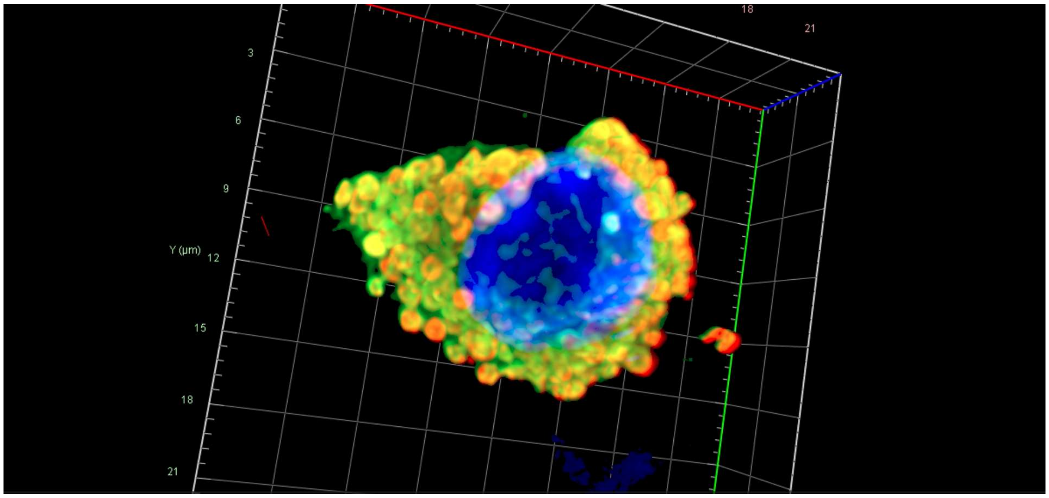

Supplementary 3B

3D model of the cytotopography of specific mast cell proteases with simultaneous content of tryptase and carboxypeptidase A3: volumetric visualization of the intracellular localization of tryptase (green color).

https://disk.yandex.ru/d/BhI7DVuhaPSZXA

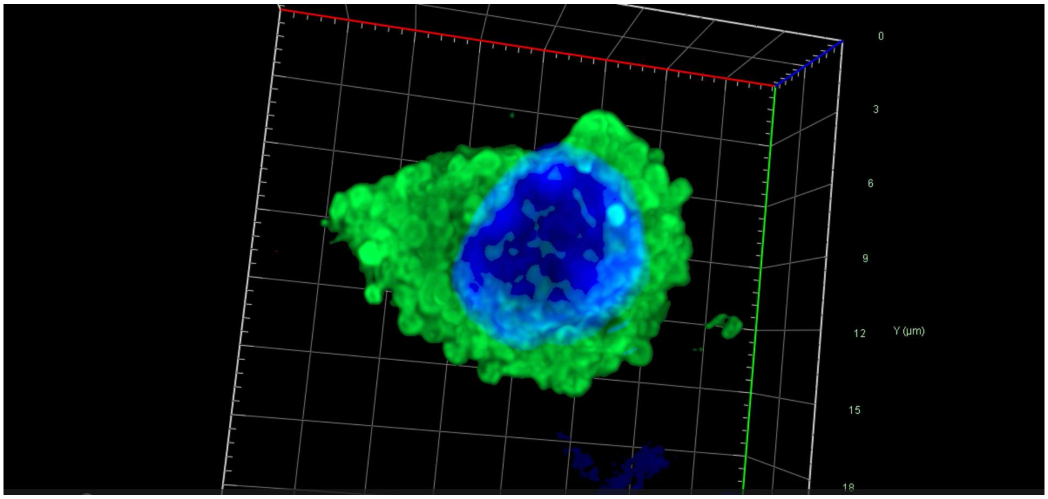

Supplementary 3C

3D model of the cytotopography of specific mast cell proteases with simultaneous content of tryptase and carboxypeptidase A3: volumetric visualization of the intracellular localization of carboxypeptidase A3 (red color).

https://disk.yandex.ru/d/eEmK2hM5hWq0sA

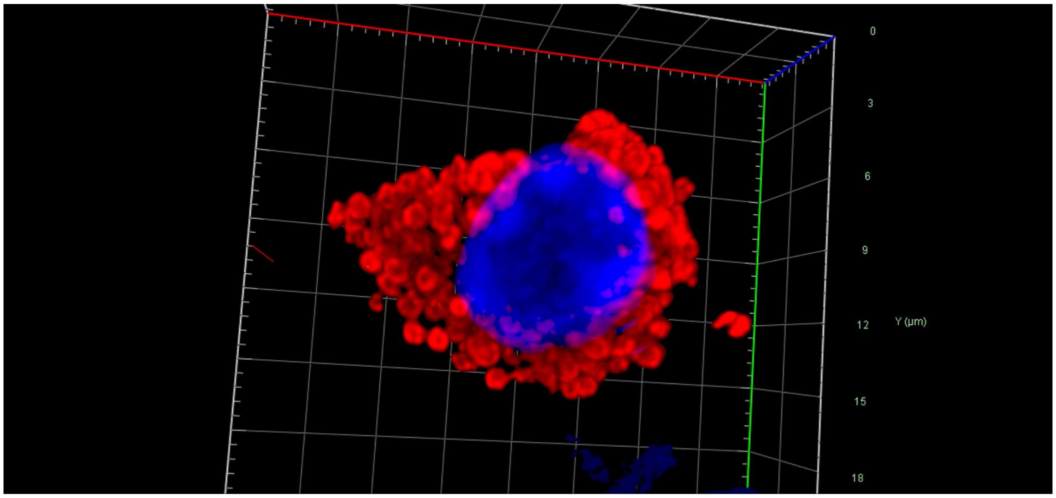

Supplementary 4

3D model of a round mast cell with the Tryptase+Chymase+ phenotype secreting single chymase-positive granules. Tryptase is green, chymase is red

https://disk.yandex.ru/d/obTeX9dti9yD9w

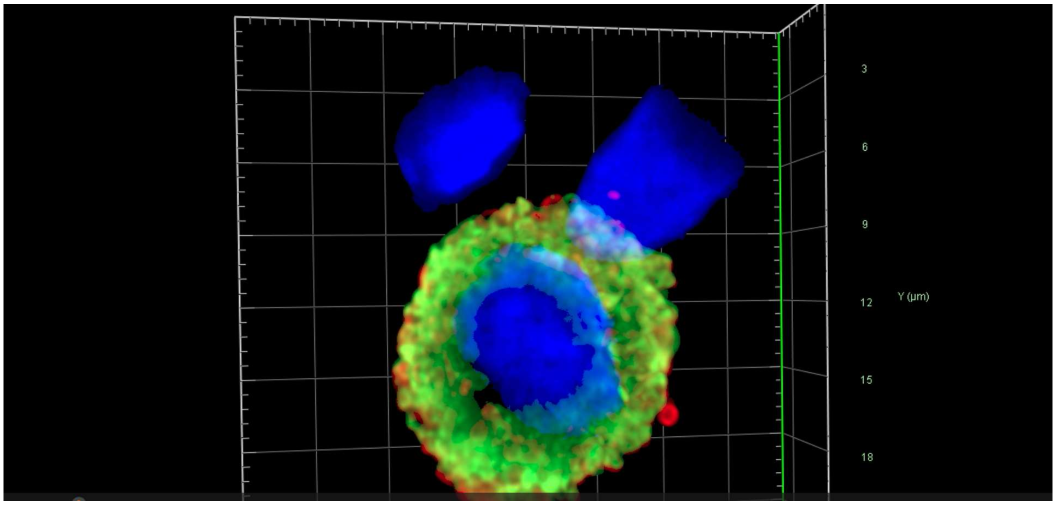

Supplementary 5

3D model of an elongated mast cell containing both tryptase (green) and chymase (red). Active secretion of granules with the Tryptase−Chymase+ and Tryptase+ Chymase+ phenotypes is observed.

https://disk.yandex.ru/d/rNbZ9JT70QuBZA

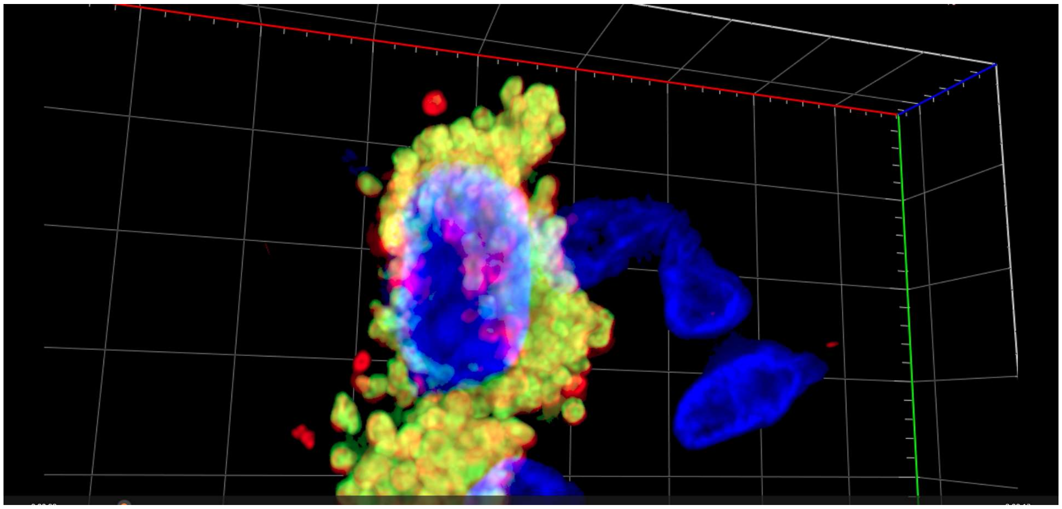

Supplementary 6

3D model of active mast cell degranulation features with transfer of autonomous secretory granules (arrowed) into the pathological osteoid area of the tumor microenvironment. Tryptase is green, chymase is red.

https://disk.yandex.ru/d/9VTWlqkVYFwKSg

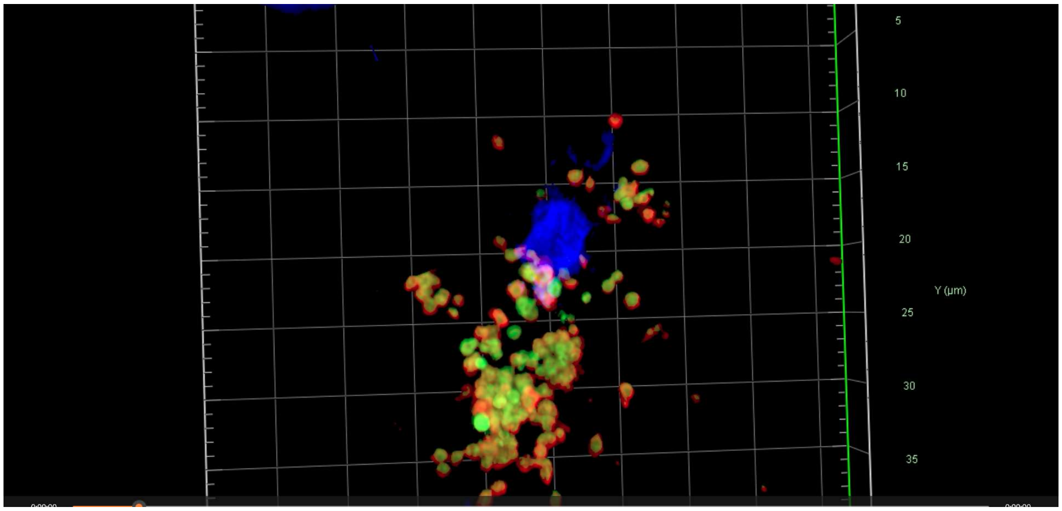

Supplementary 7

The predominant mechanism of transgranulation in the tryptase (green) and chymase (red) delivery to tumor microenvironment targets, with preserving secretion of individual granules into the extracellular matrix.

https://disk.yandex.ru/d/pL46lROWVZEeWQ

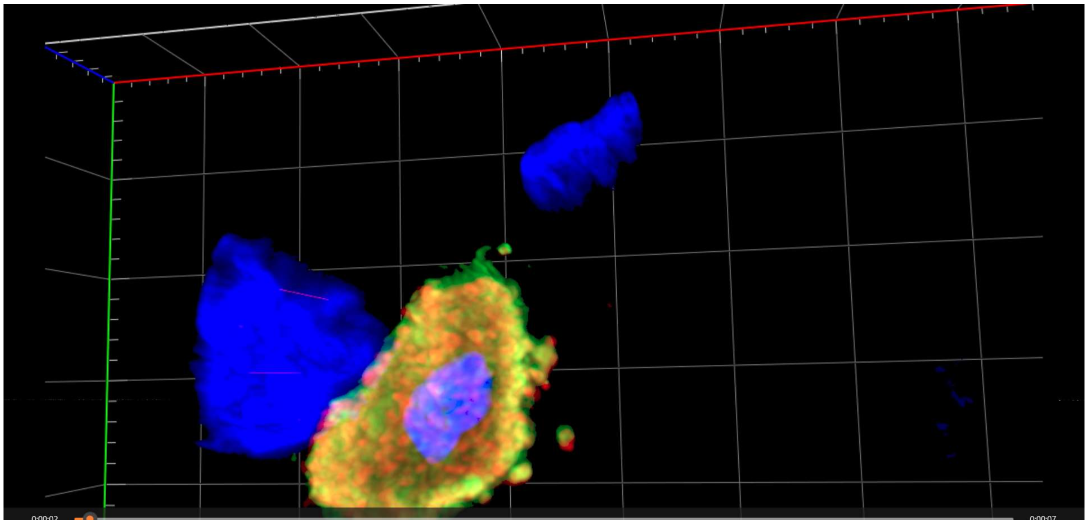

Supplementary 8

3D model of pronounced mast cell degranulation with dissemination of secretory granules of various phenotypes in the area of pathological osteoid, including Tryptase+CPA3+, Tryptase+CPA3- and tryptase-CPA3+. Tryptase is green, CPA3 is red.

https://disk.yandex.ru/d/f9jyqvenax8pqQ

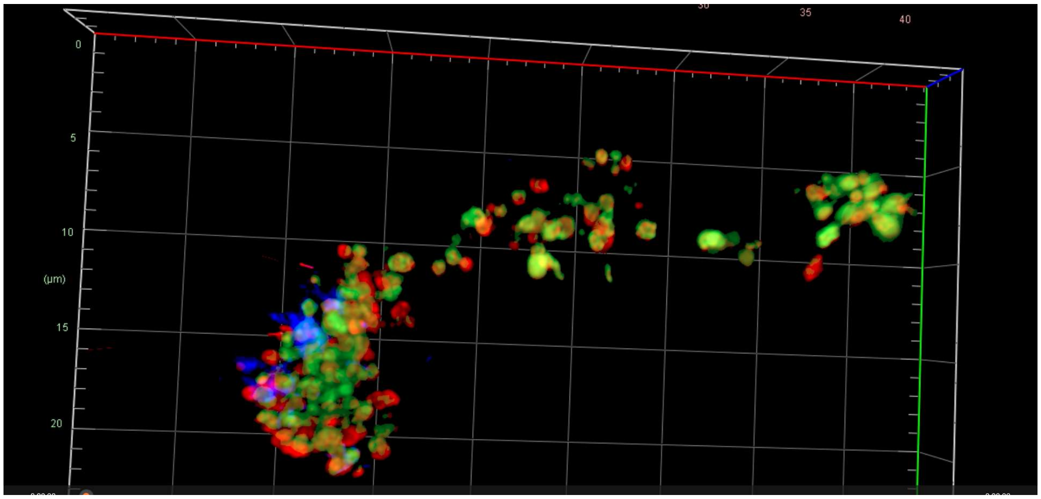

Supplementary 9

3D model of juxtacrine interaction between mast cells (green) and CD3+ lymphocytes (red).

https://disk.yandex.ru/d/kqPa1989kswWvw

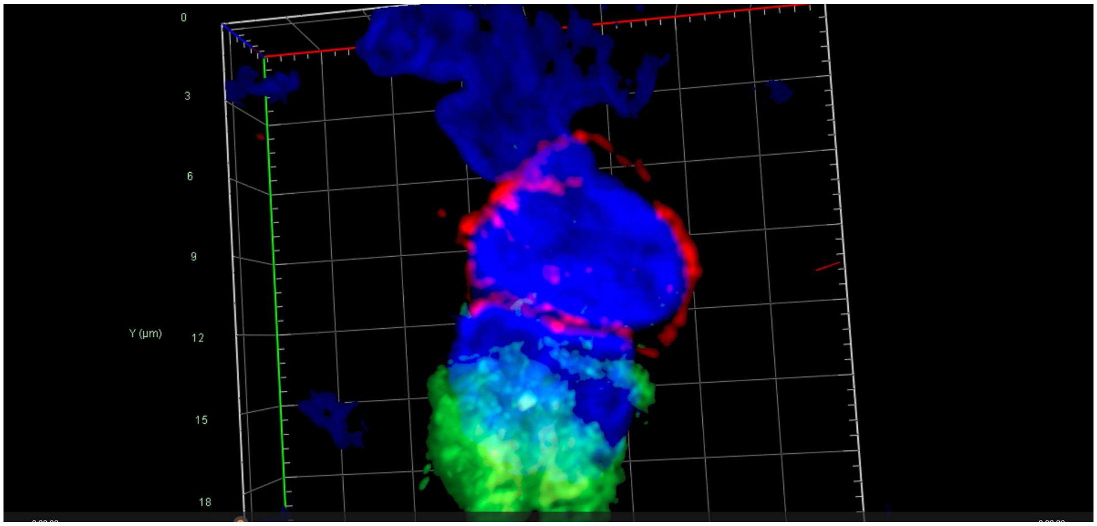

Supplementary 10

3D model of MC (green) and CD8+ lymphocyte (red) direct contacting.

https://disk.yandex.ru/d/NI4xnjiqUDYq_Q

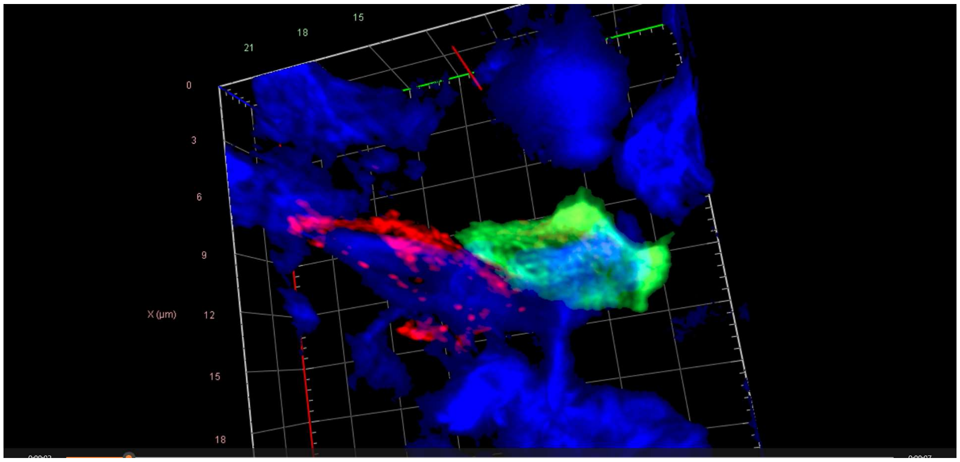

Supplementary 11

3D model of paracrine colocalization of a MC (green color) and a T-killer (red color).

https://disk.yandex.ru/d/LOHRboy7Mz-5bA

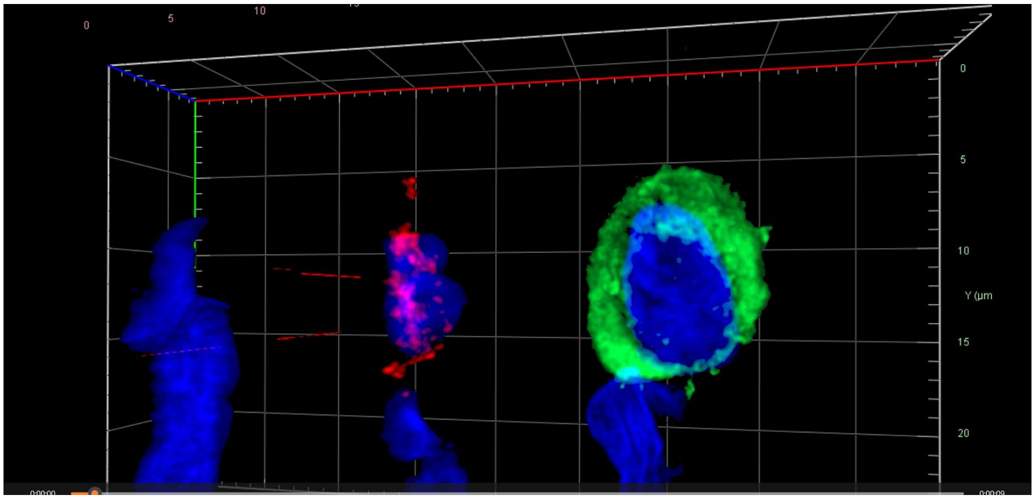

Supplementary 12

3D model of close MC colocalization (green) with endothelium (red) and signs of tryptase secretion to the basement membrane and cytoplasm of endothelial cells.

https://disk.yandex.ru/d/kuzeiaNRByFl0A

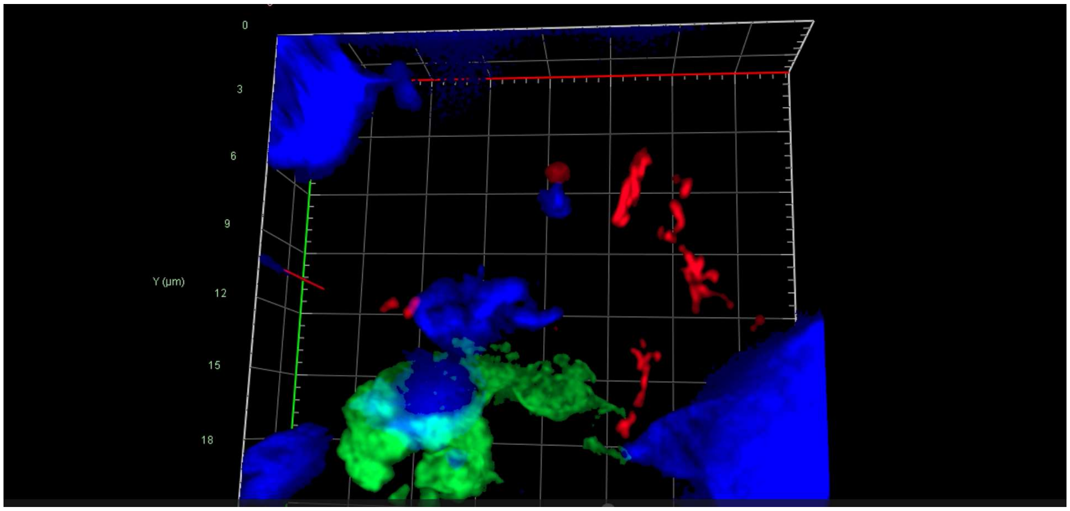

Supplementary 13

3D model of contacting between MC (green) and M2 type macrophage (red) https://disk.yandex.ru/i/NxXJC7zWwTl6yg

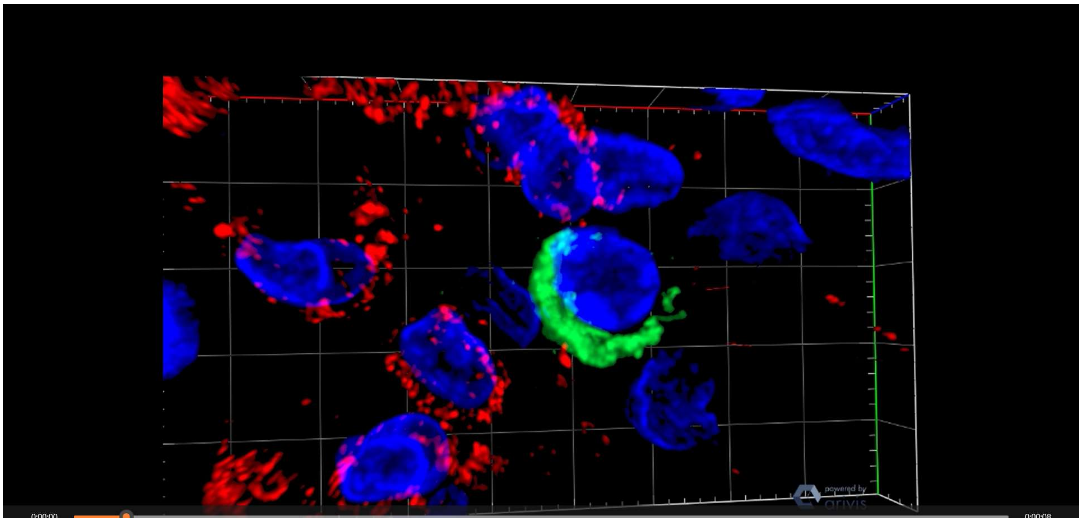

Supplement: Supplementary file 1 [file medsci-13-00195-s001.zip › medsci-3833758-supplementary.pdf]
